# A Single Amino Acid Change in the Marburg Virus Glycoprotein Arises during Serial Cell Culture Passages and Attenuates the Virus in a Macaque Model of Disease

**DOI:** 10.1128/mSphere.00401-17

**Published:** 2018-01-03

**Authors:** Kendra J. Alfson, Laura E. Avena, Jenny Delgado, Michael W. Beadles, Jean L. Patterson, Ricardo Carrion, Anthony Griffiths

**Affiliations:** a Department of Virology and Immunology, Texas Biomedical Research Institute, San Antonio, Texas, USA; b University of Texas Health Science Center, San Antonio, Texas, USA; Boston University School of Medicine

**Keywords:** Marburg virus, adaptation, attenuation, glycoprotein, monkey model, pathogenesis, signal peptide

## Abstract

Marburg virus (MARV) causes disease with high case fatality rates, and there are no approved vaccines or therapies. Licensing of MARV countermeasures will likely require approval via the FDA’s Animal Efficacy Rule, which requires well-characterized animal models that recapitulate human disease. This includes selection of the virus used for exposure and ensuring that it retains the properties of the original isolate. The consequences of amplification of MARV for challenge studies are unknown. Here, we serially passaged and characterized MARV through 13 passes from the original isolate. Surprisingly, the viral genome was very stable, except for a single nucleotide change that resulted in an amino acid substitution in the hydrophobic region of the signal peptide of the glycoprotein (GP). The particle/PFU ratio also decreased following passages, suggesting a role for the amino acid in viral infectivity. To determine if amplification introduces a phenotype in an animal model, cynomolgus macaques were exposed to either 100 or 0.01 PFU of low- and high-passage-number MARV. All animals succumbed when exposed to 100 PFU of either passage 3 or 13 viruses, although animals exposed to the high-passage-number virus survived longer. However, none of the passage 13 MARV-exposed animals succumbed to 0.01-PFU exposure compared to 75% of passage 3-exposed animals. This is consistent with other filovirus studies that show some particles that are unable to yield a plaque in cell culture can cause lethal disease *in vivo*. These results have important consequences for the design of experiments that investigate MARV pathogenesis and that test the efficacy of MARV countermeasures.

**IMPORTANCE** Marburg virus (MARV) causes disease with a high case fatality rate, and there are no approved vaccines or therapies. Serial amplification of viruses in cell culture often results in accumulation of mutations, but the effect of such cell culture passage on MARV is unclear. Serial passages of MARV resulted in a single mutation in the region encoding the glycoprotein (GP). This is a region where mutations can have important consequences on outbreaks and human disease [S. Mahanty and M. Bray, Lancet Infect Dis 4:487–498, 2004, https://doi.org/10.1016/S1473-3099(04)01103-X]. We thus investigated whether this mutation impacted disease by using a cynomolgus macaque model of MARV infection. Monkeys exposed to virus containing the mutation had better clinical outcomes than monkeys exposed to virus without the mutation. We also observed that a remarkably low number of MARV particles was sufficient to cause death. Our results could have a significant impact on how future studies are designed to model MARV disease and test vaccines and therapeutics.

## Abstract (precis)

Marburg virus (MARV) causes disease with a high case fatality rate, and there are no approved vaccines or therapies. Serial amplification of viruses in cell culture often results in accumulation of mutations, but the effect of such cell culture passage on MARV is unclear. Serial passages of MARV resulted in a single mutation in the region encoding the glycoprotein (GP). This is a region where mutations can have important consequences on outbreaks and human disease [S. Mahanty and M. Bray, Lancet Infect Dis 4:487–498, 2004, https://doi.org/10.1016/S1473-3099(04)01103-X]. We thus investigated whether this mutation impacted disease by using a cynomolgus macaque model of MARV infection. Monkeys exposed to virus containing the mutation had better clinical outcomes than monkeys exposed to virus without the mutation. We also observed that a remarkably low number of MARV particles was sufficient to cause death. Our results could have a significant impact on how future studies are designed to model MARV disease and test vaccines and therapeutics.

## INTRODUCTION

Marburg virus (MARV) is a negative-sense, single-stranded, nonsegmented RNA virus that belongs to the family *Filoviridae*. Marburg virus can cause severe disease with high case fatality rates (1, 2). No approved vaccines or therapies exist for MARV infections, despite urgent need. However, advancements in countermeasure development are hampered by the need to work with this virus in maximum containment (biosafety level 4 [BSL4]). Evidence suggests that fruit bats are the natural reservoir for MARV (3–5), but filoviruses can also replicate in other hosts, including humans, nonhuman primates (NHPs), and pigs (6, 7). Indeed, the western Africa outbreak that became the largest Ebola virus (EBOV) disease outbreak on record in 2014 highlights the capacity for filoviruses to emerge and cause a global threat and the urgency for advancements in this area (8).

The development and testing of vaccines or therapeutics against MARV require excellent animal models and a thorough understanding of those models. This is especially true given that such studies may be subject to the U.S. Food and Drug Administration’s (FDA’s) Animal Efficacy Rule (21 CFR 314.600 and 601.90), which permits the substitution of animals for humans in efficacy trials of countermeasures against highly lethal pathogens (9, 10). However, some historical studies have been performed with viruses of uncertain provenance (e.g., unknown passage history). This is important because it is known that amplification of other filoviruses, including EBOV and Sudan virus (SUDV), results in genetic changes that impact pathogenesis in animal models (11–13). However, the effect of amplification of MARV on pathogenesis is unknown. Furthermore, one of the more notable changes found after cell culture passage of EBOV and SUDV occurs at the glycoprotein (GP) RNA editing site (11, 12). However, MARV does not contain this editing site, so it is difficult to predict how cell culture passages might affect expression of GP in MARV.

Cell culture passaging has also been shown to introduce phenotypic changes that may all have impacts on animal studies. For example, specific infectivity increases when EBOV is passaged in cell culture (14). Specific infectivity can be described as the total number of viral particles present for every one particle that is able to generate a plaque in a cell culture. This is often represented as the ratio of particles per PFU (particle/PFU ratio): a lower ratio means more viral particles yield plaques in cell culture (15, 16). This ratio has been shown to impact virulence in an animal model of EBOV exposure (14).

Herein, we tested the consequences of serial passages of MARV (Angola variant) and determined if this influenced virulence in the cynomolgus macaque model of MARV infection. We also used this model of MARV infection to investigate the consequences of exposure to a low dose of virus.

## RESULTS

### Changes to genotype as Marburg virus is passaged in Vero E6 cell culture.

**FIG 1:**
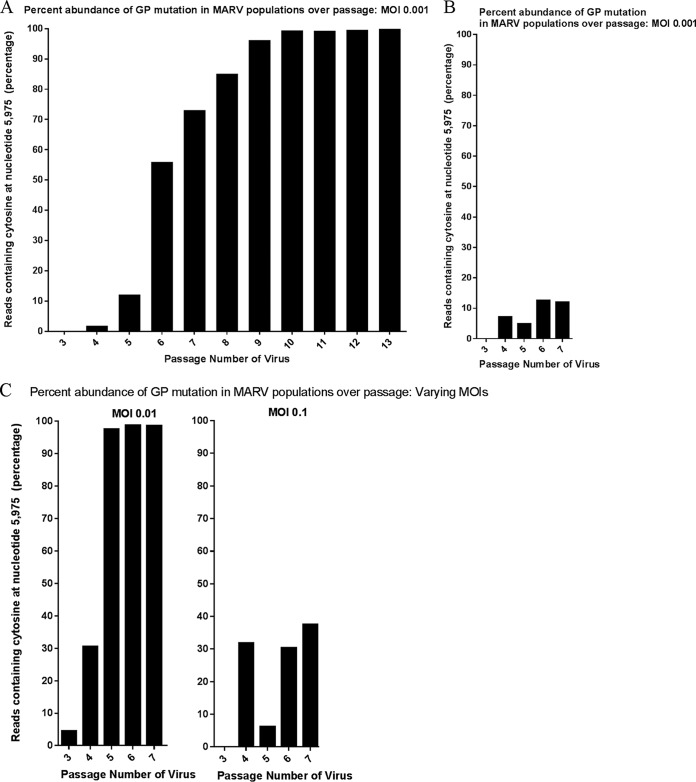
Percentage of abundance of reads containing SNP in the MARV GP gene in populations over passage in Vero E6 cell culture. MARV was serially passaged in Vero E6 cells at three different MOI (0.001, 0.01, and 0.1). Deep sequencing was used to investigate genotypic changes that occurred after cell culture passaging. Graphs display the relative abundance of reads containing cytosine (C) at the SNP locus site, after each passage. (A) MARV was passaged 10 times at an MOI of 0.001. (B) The first five passages were repeated at the same MOI (0.001). (C) The first five passages were repeated at two different higher MOI (0.01 and 0.1).

To determine if MARV undergoes genotypic changes during cell culture passage, we serially passaged MARV (Angola variant) 11 times in Vero E6 cells at a multiplicity of infection (MOI) of 0.001 PFU/cell. The starting material was passage 2 (P2) MARV, previously isolated from a fatal human case and then passaged independently twice in Vero E6 cells at an unknown MOI). The cells, virus, and passage conditions were selected based on recommendation of the Filovirus Animal Nonclinical Group (FANG), which contains representatives from various stakeholders who have an interest in developing licensed countermeasures for filovirus infections (17). After each passage, the viral titer was determined and the amplification was repeated; whole-genome sequencing was used to investigate genomic changes. Passages of MARV resulted in only a single nucleotide (nt) change to the consensus sequence (present in greater than 50% of reads) between the P3 virus (median depth of coverage of 3,500 nt and mean depth of coverage of 3,515 nt) and P13 virus (median depth of coverage of 21,808 nt and mean depth of coverage of 22,185 nt) (Fig. 1A). For P3 virus, 100% of reads contained uracil (U) at nt 5975 (amino acid 12) in the GP gene (MARV_gp4), while for P13 virus, 99.5% of reads contained cytosine (C) at the same locus (nt 5975 and amino acid 12). This single nucleotide polymorphism (SNP) causes a leucine-to-serine change in the signal peptide region. The abundance of this SNP increased following passages in Vero E6 cells; the mutation reached consensus levels at passage 6 (Fig. 1A). To determine if appearance of the SNP was reproducible, serial passage was repeated. When the P2 virus was serially passaged again (five times at an MOI of 0.001 in Vero E6 cells), the SNP appeared at passage 4 and increased over passage, but less rapidly than in the first replicate (Fig. 1B). When serial passage was performed at an MOI of 0.01, the SNP reached a consensus level after passage 4, and when an MOI of 0.1 was used, the SNP increased in abundance but did not reach a consensus level (Fig. 1C).

### Changes to particle/PFU ratio as Marburg virus is passaged in Vero E6 cell culture.

**FIG 2:**
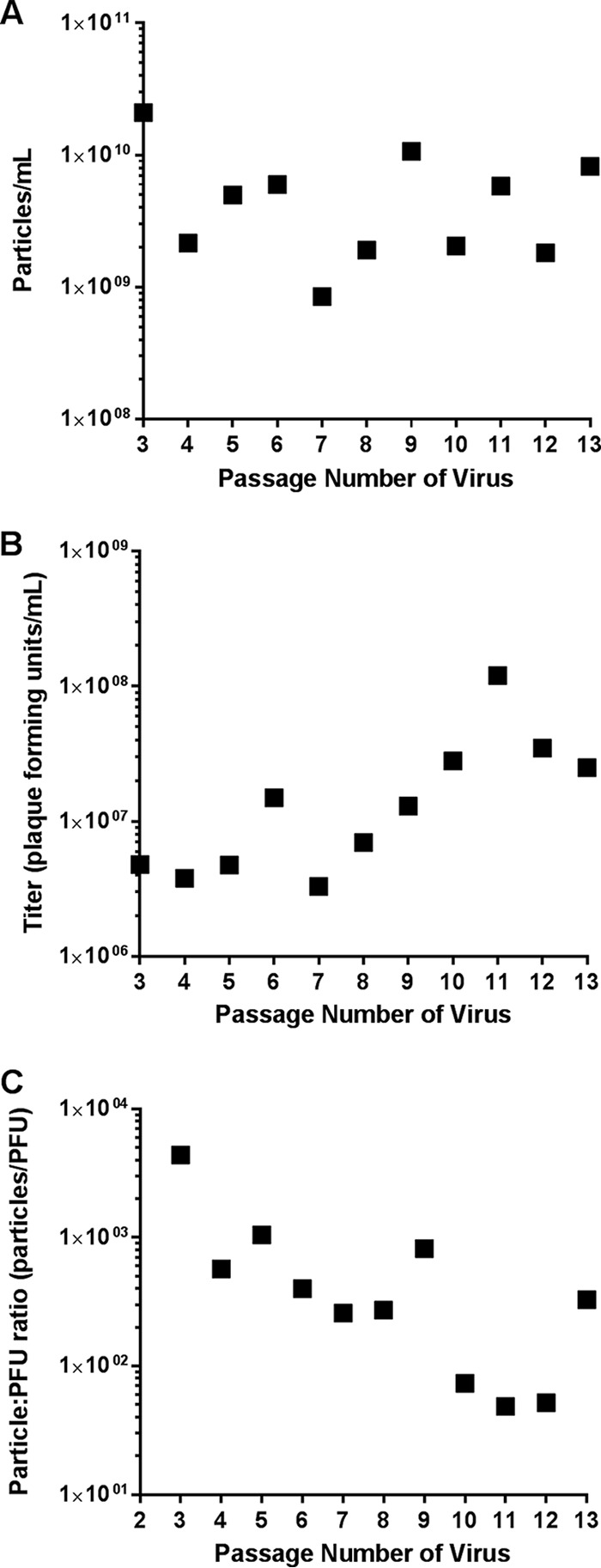
Changes in MARV particle/PFU ratio after cell culture passage in Vero E6 cells. Passage 2 MARV was serially passaged 10 times in Vero E6 cells at an MOI of 0.001. (A) After each passage, the number of virus particles per milliliter was determined via TEM (singlicate). (B) After each passage, the viral titer (PFU per milliliter) was determined via plaque assay (singlicate). (C) After each passage, the particle/PFU ratio was determined based on the titer and number of particles.

To determine the specific infectivity of MARV in Vero E6 cells, after each passage the titer and the concentration of particles were determined and the particle/PFU ratio was calculated. As shown in Fig. 2A, low-passage-number MARV stocks cultured in Vero E6 cells yielded high concentrations of particles (2× 10^9^ to 2× 10^10^ particles/ml). The titers were 3 to 4 logs lower (3 × 10^6^ to 4 × 10^6^ PFU/ml) than the particle numbers (Fig. 2B), resulting in particle/PFU ratios of approximately 5 × 10^2^ to 5 × 10^3^ particles/PFU. As the virus was passaged, we observed an increase in the concentration of Vero E6 cell infectious virus particles, while the concentration of total particles in the supernatant remained relatively constant. The last passages had particle/PFU ratios 1 to 2 logs lower than the first passages (Fig. 2C). Thus, serial passage in Vero E6 cells correlated with a decrease in particle/PFU ratio.

### Effect of MARV cell culture passaging during nonhuman primate infection.

To test the hypothesis that low-passage-number MARV stocks have increased potency relative to high-passage-number stocks, the virulence of two virus stocks was determined using the cynomolgus macaque model of MARV infection (Table 1). Cynomolgus macaques were exposed intramuscularly (i.m.) to one of two different doses (either 0.01 or 100 PFU) of MARV stock that was either low passage number (P3; *n =* 8) or high passage number (P13; *n =* 8). After each dose was prepared for the NHP exposure, the concentration of Vero E6 cell infectious particles present was determined using a plaque assay. For the group that received a target dose of 100 PFU, the P3 dose was determined to contain 21 PFU and the P13 dose was determined to contain 81 PFU. The low dose (0.01 PFU) did not contain sufficient Vero E6 cell infectious particles to accurately quantify using a plaque assay. However, as determined by whole-genome sequencing, serum collected at or near time of death exhibited evidence of viral RNA in all except one animal, consistent with exposure to infectious virus. Survival and other clinical observations are described below.

**TABLE 1:** Study design

MARV passage	Target dose (PFU)	Particle count (particles/PFU)	No. of particles in target dose	Dose delivered (PFU)	Cohort size	Survival
P3	100	1 × 10^3^	1 × 10^5^	21	4	0/4
	0.01	1 × 10^3^	10	0.0021	4	1/4
P13	100	3 × 10^2^	3 × 10^4^	81	4	0/4
	0.01	3 × 10^2^	3	0.0081	4	4/4

### (i) Survival.

**FIG 3:**
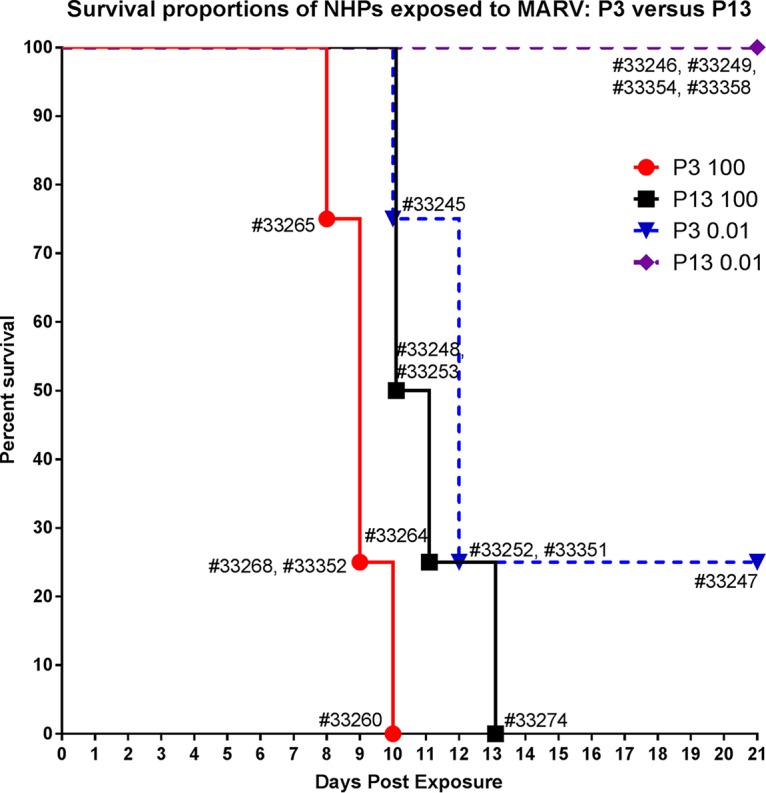
Survival proportions of NHPs exposed to MARV. Shown are survival proportions of animals exposed to either 100 or 0.01 PFU of MARV that was either low passage number (P3) or high passage number (P13). Results from individual animals are displayed.

Figure 3 shows the survival proportions of NHPs exposed to either 100 or 0.01 PFU of low- or high-passage-number MARV. All eight NHPs exposed to 100 PFU died between 8 and 13 days postexposure (median survival times of 9 days postexposure for the P3 group and 10.5 days postexposure for the P13 group). Three of four animals exposed to 0.01 PFU of P3 virus succumbed (median survival of 12 days postexposure). No animals exposed to 0.01 PFU of P13 virus succumbed. There was a statistically significant difference in survival between the P3 and P13 groups following exposure to both 100 PFU (*P* = 0.031, Mantel-Cox test) and 0.01 PFU (*P* = 0.042, Mantel-Cox test).

### (ii) Viremia.

**FIG 4:**
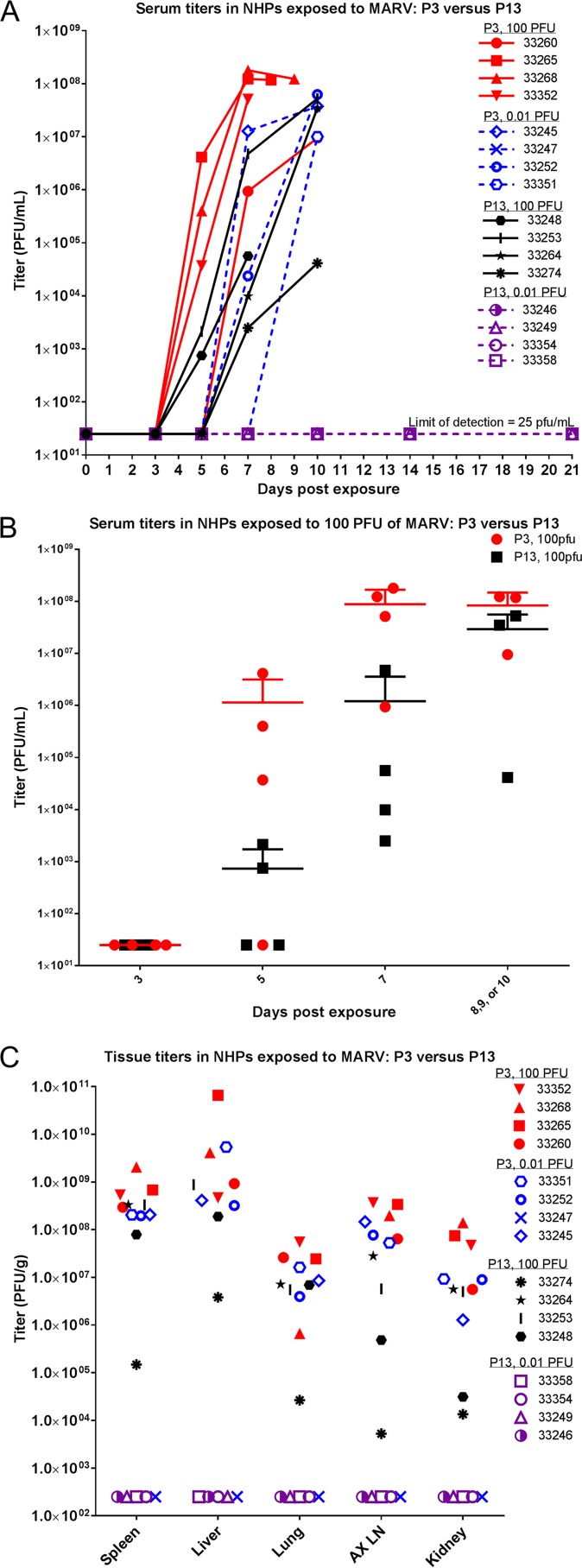
Serum and tissue titers in NHPs exposed to MARV. (A) At each scheduled sedation, blood was collected from all NHPs, and serum was later processed to determine levels of infectious virus in the blood. Serum titers (PFU per milliliter) for all NHPs throughout the course of the study are shown. (B) Serum titers (PFU per milliliter) for animals exposed to the 100-PFU group of either P3 or P13 virus are shown. Means with standard deviations (SD) are shown. (C) When animals were necropsied, samples of tissue were taken and later analyzed for viral load. Titers (PFU per gram) from selected tissues for all NHPs are shown.

Viremia was examined via plaque assay using serum collected on scheduled blood collection dates, serum collected at or near time of death, and tissues collected during necropsy (Fig. 4). All but one NHP that succumbed began exhibiting viremia between days 5 and 7 postexposure (NHP 33351 did not exhibit viremia until the day of death, 10 days postexposure). All NHPs that succumbed (all NHPs exposed to 100 PFU and three NHPs exposed to 0.01 PFU of P3 virus) had evidence of infectious virus in serum on the day of death (or on the day closest to death for those found dead). Conversely, NHPs that survived to the scheduled project end (one NHP exposed to 0.01 PFU of P3 virus and all NHPs exposed to 0.01 PFU of P13 virus) did not exhibit measurable levels of infectious virus throughout the course of the study. Figure 4B compares the serum titers (in PFU per milliliter) of P3- versus P13-exposed NHPs in the 100-PFU group. These data show that there was a delay in reaching high serum titers for NHPs exposed to P13 virus. When comparing serum titers of NHPs exposed to P3 versus P13 virus, significant differences were not found on days 3 or 5 postexposure or near time of death (*P* > 0.05); only day 7 postexposure serum titers exhibited a significant difference (*P* < 0.01) (two-way analysis of variance [ANOVA] Bonferroni posttest). Figure 4C shows tissue titers (PFU per gram) for all NHPs at time of death. Nonhuman primates exposed to 0.01 PFU of P13 virus did not exhibit measureable levels of infectious virus in any of the tissues tested. In general, NHPs exposed to the P3 virus exhibited higher levels of virus in the tissues tested. Titers were highest in liver and spleen.

### (iii) Clinical observations.

**FIG 5:**
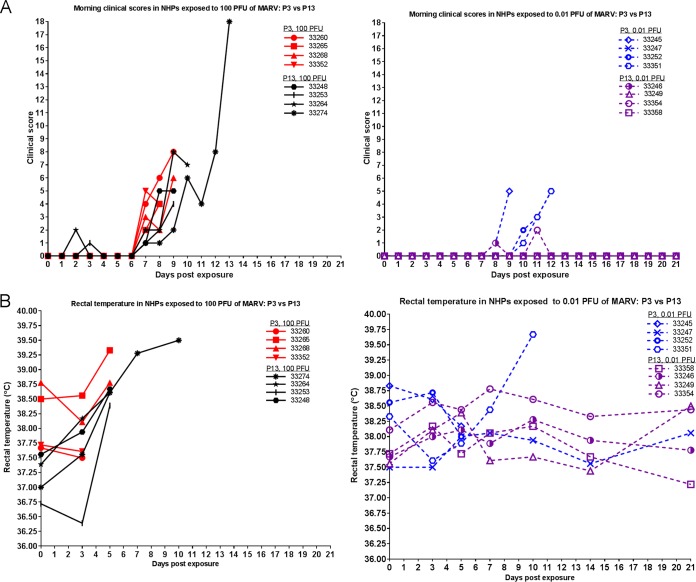
Clinical scores and temperature in MARV-exposed NHPs. (A) Animals were observed at least twice daily, and clinical scores were recorded. Clinical scores from the morning observation periods for all NHPs throughout the course of the study are shown. (B) At each scheduled sedation, rectal temperatures (°C) were taken. Rectal temperatures for all NHPs throughout the course of the study are shown.

Figure 5A shows the clinical scores taken during morning observations for all NHPs throughout the course of the study. (Clinical scores were recorded up to four times per day.) Early signs of decreased food consumption (greater than 75% decrease) and reduced fluid intake preceded the expected reduced stool production for most NHPs succumbing to infection. Additional clinical observations included increases in body temperature and mild to severe petechia. For NHPs that succumbed, there was not a considerable difference in scores between the P3 and P13 groups, with the exception of NHP 33274 (in the P13 100-PFU group), which had the highest score of all NHPs. (This could be related to a longer survival time prior to succumbing to disease.) Within each group, NHPs exposed to 0.01 PFU showed a delay in exhibiting scores and generally had lower scores. Another important parameter in the progression of MARV disease is febrility (Fig. 5B). As the disease progressed, many NHPs became febrile. For NHPs that succumbed, there was not a marked difference in temperature data.

**FIG 6:**
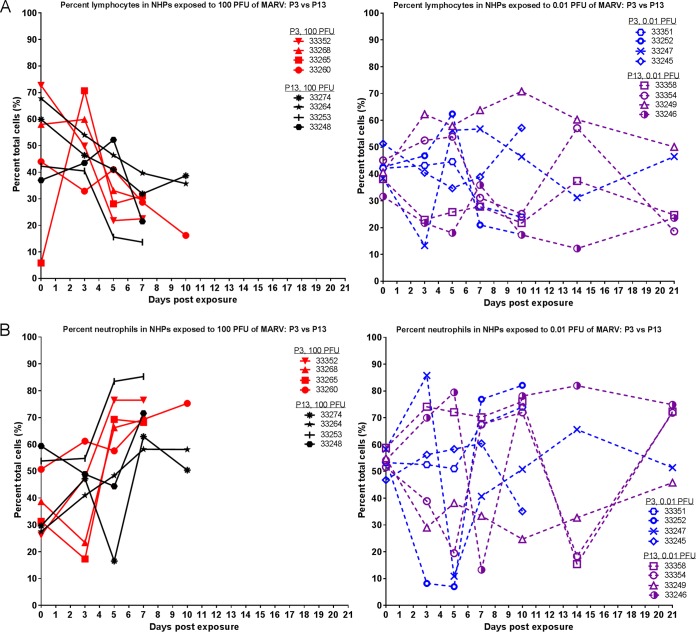
Lymphocyte and neutrophil percentages in MARV-exposed NHPs. During each scheduled sedation, blood specimens were collected and analyzed. Results of hematology analysis for all NHPs throughout the course of the study are shown. (A) Percentage of lymphocytes. (B) Percentage of neutrophils.

Complete blood counts, chemistry analyses, and coagulation tests were performed on blood collected throughout the study. Observations in the NHPs that succumbed were consistent with filovirus infection in experimentally infected NHPs, as previously described (12, 18–20). Figure 6 shows lymphocyte and neutrophil percentages throughout the duration of the study. Those that succumbed showed a relative reduction of lymphocytes and an increase in neutrophils; there was no significant difference between the P3 and P13 groups.

**FIG 7:**
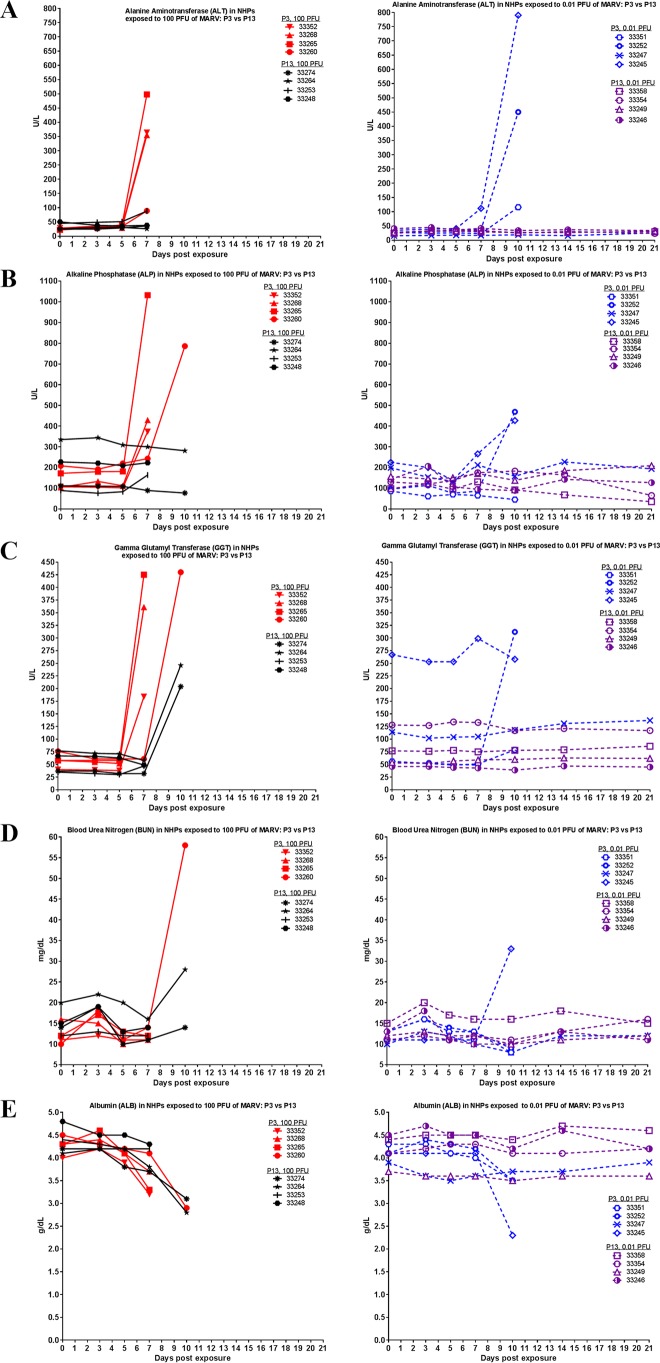
Clinical chemistry parameters in MARV-exposed NHPs. During each scheduled sedation, blood specimens were collected and analyzed. Results of clinical chemistry analysis for all NHPs throughout the course of the study are shown. (A) ALT. (B) ALP. (C) GGT. (D) BUN. (E) ALB.

As seen in Fig. 7, increases in clinical chemistry parameters indicative of liver damage (increases in alanine aminotransferase [ALT], alkaline phosphatase [ALP], and gamma glutamyl transferase [GGT]) were observed just prior to death in NHPs that succumbed. Survivors did not exhibit the same marked increases. The increase was less pronounced in NHPs exposed to P13 versus P3 virus. Values for ALT, ALP, and GGT are shown in Fig. 7. Increased blood urea nitrogen (BUN) and decreased albumin (ALB) are also indicative of filovirus disease in macaques (Fig. 7) (12, 19). Differences in these values between P3 and P13 groups were less pronounced, with only a few NHPs exhibiting increased BUN and most NHPs that succumbed exhibiting decreased ALB. Similar to what we observed with serum titers, the effect of group (P3 versus P13 virus) on variance was statistically significant for blood chemistry values (two-way ANOVA Bonferroni posttest). Within the 100-PFU group, there was a difference for ALT (*P* < 0.0001), ALP (*P* = 0.0197), and GGT (*P* = 0.0002) on day 7 postexposure. Within the 0.01-PFU group, there was a difference for ALT (*P* = 0.0020) and ALB (*P* = 0.0437) on day 10 postexposure.

**FIG 8:**
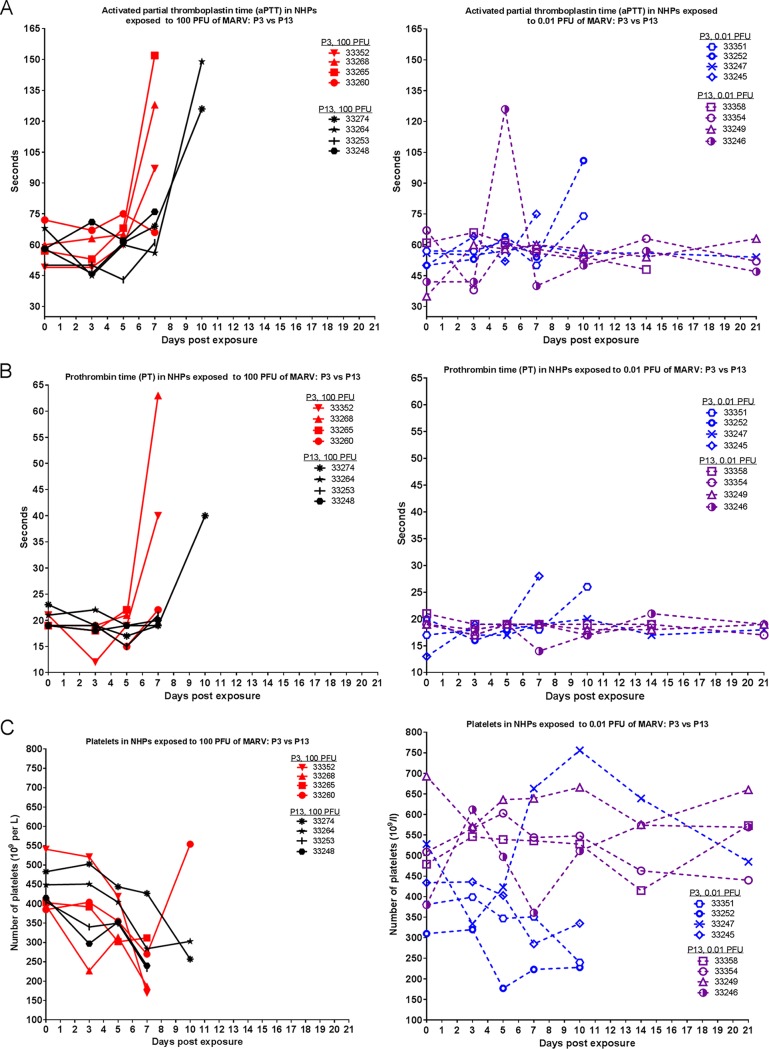
Coagulation parameters in NHPs exposed to MARV. During each scheduled sedation, blood specimens were collected and analyzed. Results of clinical coagulation analysis for all NHPs throughout the course of the study are shown. (A) PT. (B) aPTT. (C) Platelets.

Figure 8 shows prothrombin time (PT), activated partial thromboplastin time (aPTT), and platelet counts throughout the course of the study. Animals that succumbed exhibited increases in clotting time, with P13-exposed NHPs having a slight delay in exhibiting this symptom. The effect of group (P3 versus P13 virus) on variance was statistically significant for aPTT (*P* = 0.0017) and PT (*P* = 0.0018; with animal 33265 excluded from analyses because the PT value was out of range on day 7 postexposure) on day 7 postexposure within the 100-PFU group (two-way ANOVA Bonferroni posttest). Nonhuman primates that succumbed also exhibited decreased platelet counts, regardless of whether they were exposed to P3 or P13 virus.

### (iv) Anatomical pathology.

All eight NHPs exposed to 100 PFU and three of four NHPs exposed to 0.01 PFU of P3 virus presented with similar gross and histologic findings characteristic of experimental acutely fatal filovirus infection (21, 22). All five surviving NHPs lacked gross and histologic changes suggestive of filovirus infection. For NHPs that succumbed, gross observations were similar between groups and included friable, pale, and/or enlarged liver (9/11), cutaneous petechial rash (8/11), enlarged spleen (6/11), urinary bladder hemorrhage (2/11), testicular hemorrhage (2/5), and hemorrhage or red discoloration in the intestinal tract (2/11). Gross changes at the injection site included mild skin inflammation at the injection site (3/11), hemorrhage at the injection site (2/11), and moderate muscle inflammation at the injection site (4/11). Findings are summarized in Table 2. Histologic findings included necrosis and lymphoid depletion of the inguinal, axillary, and/or mediastinal lymph nodes, necrosis and lymphoid depletion in white pulp of the spleen, lymphoid depletion along with fibrin in the red pulp of the spleen, liver necrosis and swelling, and mild to moderate tubular degeneration in the kidneys.

**TABLE 2:** Gross pathology observations

MARV passage	Dose (PFU)	No. of NHPs/cohort with:				
		Petechia	Friable, pale, and/or enlarged liver	Intestinal red discoloration or hemorrhage	Testicular hemorrhage	Enlarged spleen
P3	100	2/4	4/4	1/4	1/2	2/4
	0.01	3/4	2/4	0/4	0/2	1/4
P13	100	3/4	3/4	1/4	1/2	3/4
	0.01	0/4	0/4	0/4	0/2	0/4

### Changes to genotype of Marburg virus during nonhuman primate infection.

Nucleic acid was isolated from serum taken at or near the time of death from each NHP, and whole-genome sequencing was used to determine the sequence of the GP signal peptide region in the viral RNA (Table 3). Viral RNA isolated from the eight NHPs exposed to P3 virus maintained the 100% U genotype, representing no change from the parent P3 virus used during exposure. Viral RNA isolated from NHPs exposed to P13 showed more variability. Two of four NHPs exposed to 100 PFU of P13 virus maintained the 100% C genotype found in the P13 parent virus used for exposure. The other two of four NHPs exposed to 100 PFU of P13 virus exhibited a mixed viral population with a U present at more than 50% abundance in the viral RNA. One of four NHPs exposed to 0.01 PFU of P13 virus did not exhibit enough coverage across the genome to determine definitively the genotype. The other three of four NHPs exposed to 0.01 PFU of P13 virus exhibited a mixed viral population with the abundance of U ranging from less than 10% to greater than 70%. There were no clear correlations between rate of disease progression or severity and virus genotype. All animals that succumbed to exposure began exhibiting clinical signs indicative of MARV exposure approximately 2 days prior to succumbing, and the signs exhibited were similar.

**TABLE 3:** Abundance of GP signal peptide SNP in serum for all MARV-exposed NHPs

Group and dose	NHP ID	% of U genotype in GP signal sequence	Depth of coverage at SNP location (no. of reads at locus)	Day of death (days postexposure)
P3				
100 PFU	33260	100	24,145	10
	33265	100	81,769	8
	33268	100	74,571	9
	33352	100	29,203	9^a^
0.01 PFU	33245	100	58,208	10
	33247	100	45	21
	33252	100	32,510	12^a^
	33351	100	5,902	12
P13				
100 PFU	33248	36	151	10^a^
	33253	<1	36,675	10
	33264	<1	33,040	11
	33274	30	779	13
0.01 PFU	33246	75	175	21
	33249	8	142	21
	33354	73	235	21
	33358	NA^b^	NA^b^	21

a Animal was found dead in cage, and thus no serum sample was available from the day of death; these results are described as being “near day of death.”

b NA, did not exhibit enough coverage across the genome to definitively determine the genotype.

Similar to what was observed during cell culture passage, MARV did not exhibit much genotypic diversity at the consensus sequence level after *in vivo* replication. Besides the aforementioned SNP in GP, nonsynonymous SNPs at the consensus sequence level were only found in viral RNA collected from the serum of two animals. Both animals were in the group exposed to 0.01 PFU of P3 virus. Viral RNA in serum collected from animal 33252 contained one SNP in GP (nt 7481, amino acid 514) that resulted in an alanine-to-valine substitution (depth of 31,865 nt at locus) and one SNP in VP35 (nt 3400, amino acid 152) that resulted in a leucine-to-phenylalanine substitution (depth of 33,825 nt at locus). Viral RNA in serum collected from animal 33245 contained two SNPs in VP40 (nt 4650, amino acid 28, and nt 5111, amino acid 182) that resulted in an isoleucine-to-threonine substitution (depth of 23,580 nt at locus) and a serine-to-proline substitution (depth of 46,048 nt at locus), respectively. The VP35 SNP (nt 3400) also appeared during the cell culture passaging experiments but only reached an abundance of 27% (at passage 5) before decreasing again and disappearing.

## DISCUSSION

Marburg virus can cause severe disease with high case fatality rates, and no approved vaccines or therapies exist to treat MARV infections. The development and testing of effective vaccines or therapeutics against MARV require excellent animal models and a thorough understanding of those models. However, historical studies may have been performed with viruses of inadequately described provenance, and it is widely accepted that cell culture passages of viruses often result in genetic and phenotypic changes (23–25). However, the effect and biological consequences of cell culture adaptation to MARV are unclear.

We serially passaged MARV in Vero E6 cells and used whole-genome sequencing to identify genomic changes. Serial passages of MARV in cell culture correlated with increased specific infectivity, but the genotype did not exhibit much diversity following passages. There was only a single nucleotide difference in the consensus sequence between the P3 and P13 viruses; this SNP occurred within the GP gene. To place this in context, guinea pig-adapted EBOV was shown to have 10 nucleotide changes in the consensus sequence from the input virus (26), and mouse-adapted MARV was shown to have either 33 (MARV-Ci67) or 75 (MARV-RAVN) nucleotide changes from each input virus (27).

To test whether this mutation had biological consequences *in vivo*, the virulence of P3 versus P13 virus was determined using a cynomolgus macaque model of MARV infection. Nonhuman primates were exposed i.m. to either 0.01 or 100 PFU of either P3 or P13 MARV. There was a statistically significant difference in median survival between the P3 and P13 groups, and there was a delay in reaching high serum titers for NHPs exposed to P13 virus. Nonhuman primates exposed to P13 virus also showed smaller increases, or delays in exhibiting increases, for blood chemistry values (ALT, ALP, and GGT) and clotting times (aPTT and PT). This suggests that the cell-culture-passaged virus may be attenuated *in vivo*. Additionally, at the low dose, cell-culture-passaged virus was infectious but not lethal, which may have allowed for viral persistence. Three of the four animals that survived exposure with 0.01 PFU of P13 virus did not exhibit overt signs of disease, yet serum from these animals contained viral RNA 21 days postexposure (as determined by whole-genome sequencing). The potential for viral persistence has also been seen during MARV outbreaks, as evidenced by the presence of viral RNA in bodily fluids after a period of convalescence (28–31).

In filoviruses, the GP gene encodes the structural surface glycoprotein that studs the envelope and is involved in attachment, fusion, tropism, virus spread, and pathogenicity (32). The SNP described herein causes an amino acid substitution within the conserved, very hydrophobic region of the GP signal peptide. Passage 3 virus encodes a hydrophobic leucine, while P13 virus encodes a serine at this locus. This mutation could disrupt the hydrophobicity of the signal peptide, which could affect function. For example, the signal peptide is responsible for trafficking of GP within the cell (33), so changes in the signal peptide could change protein trafficking. Indeed, the particle/PFU ratio decreased concomitantly with appearance of the amino acid change, implying that the amino acid has a role in viral infectivity. Perhaps the P13 genotype (C) confers increased efficiency in virus assembly, thus leading to enhanced production of infectious virions. That this genotype was also associated with decreased virulence suggests that such increased efficiency is cell culture specific and the SNP does not provide the same advantage *in vivo*.

Furthermore, for EBOV and SUDV, one of the better studied genetic changes that results from cell culture passage is in the glycoprotein RNA editing site (11, 12). *Marburgviruses* lack this editing site, and there is less information describing how *Marburgviruses* might regulate GP expression or how cell culture passages might affect expression. For EBOV and SUDV, the genome sequence encodes a truncated, secreted form of GP (sGP), but RNA editing also allows for translation of full-length GP (34, 35). The various forms of GP and their ratios seem to play a critical role in pathogenicity (36, 37). The involvement of the genome sequence in GP regulation is likely important for the *Ebolaviruses*, and amplification *in vitro* and *in vivo* has been shown to cause changes in the genomic sequence at the editing site (11, 12, 35). However, MARV has not been shown to generate multiple products from the GP gene, and regulation of MARV GP is poorly understood. It is possible that this GP mutation could be involved in regulation of GP production. Further characterization of this mutation will help enhance our understanding of MARV GP regulation and of the MARV GP signal peptide.

As the GP mutation was the only consensus sequence change between the two viruses, it seems likely that the mutation is involved in the biological consequences. Furthermore, in the group exposed to 0.01 PFU of P13 virus, it seems the virus may have spontaneously mutated to the P3 genotype. The 0.01-PFU group received a very low quantity of viral particles (a target dose of fewer than 10 particles), and the P13 virus population (C genotype) contained less than 1% reads with a U genotype, so it seems unlikely the P13 0.01-PFU group was exposed to viral genomes that possessed the P3 genotype (U). Thus, rather than these NHPs exhibiting reversion or an increase in abundance of existing U genotype due to increased viral fitness, the mutation from C to U in the P13 group may have appeared spontaneously, suggesting a propensity toward the original hydrophobic amino acid.

The P13 MARV did exhibit increased specific infectivity, so it is possible that the attenuation seen *in vivo* with P13 virus is a result of a lower overall particle count in the exposure dose, similar to what has been seen with EBOV (14). However, it seems that a remarkably low number of MARV particles is sufficient to cause virulence. Previous work with Ebola virus suggested that approximately 80 particles was enough to cause 100% mortality in NHPs but that approximately five particles was not (14). Thus, based on the present study, MARV may be even more potent than EBOV in the cynomolgus macaque model. Furthermore, the difference in particle/PFU ratios between the two stocks used during virus exposure is small. Within the 0.01-PFU group, the P3 group was exposed to a target dose of less than 10 particles and the P13 group was exposed to a target dose of approximately 3 particles; the determined dose based on back titer of the 100-PFU dose decreases the difference more, with the P3 group being exposed to a dose of approximately 2 particles and the P13 group being exposed to a dose of approximately 3 particles. Thus, it seems unlikely that particle/PFU ratio is the major contributor to attenuation.

Due to the significance of GP and its assorted functions, mutations in this region have the potential to have significant consequences. Research has shown the Ebola virus GP is important for cell entry, and mutations can impact tropism and entry efficiency (38, 39). The phenotypic effects of mutations may also have important consequences for outbreaks and human disease (40). Finally, the target of the protective immune response during MARV infection would likely be GP, and other mutations in GP have been hypothesized to lead to immune evasion by MARV (41), thus increasing the potential importance of the mutation described herein.

Due to its reproducible appearance after cell culture passage and the potential for *in vivo* biological consequences, further investigation into this MARV GP mutation will be informative for future viral challenge stock characterization studies and *in vivo* studies of countermeasure development or disease modeling.

## MATERIALS AND METHODS

### Ethics statement, clinical scoring system, and euthanasia criteria.

Animal research was conducted under a Texas Biomedical Research Institute Institutional Animal Care and Use Committee-approved protocol (protocol 1381) in compliance with the Animal Welfare Act and other federal statutes and regulations relating to animals and experiments involving animals. The facility where this research was conducted is accredited by the Association for Assessment and Accreditation of Laboratory Animal Care International and adheres to principles stated in the 8th edition of the *Guide for the Care and Use of Laboratory Animals* from the National Research Council (42). Nonhuman primates were single housed and fed monkey biscuits. Enrichment included commercial toys and dietary enrichment. Marburg virus exposure occurred on study day zero. Nonhuman primates were then observed at least twice daily for up to 21 days postexposure, at which time the survivors were euthanized for tissue collection. During blood collections, NHPs were anesthetized using tiletamine HCl and zolazepam HCl (Zoetis), and euthanasia criteria were developed to minimize undue pain and distress. Nonhuman primates were euthanized with an intravenous overdose of sodium pentobarbital.

Clinical scores for the NHPs were reported to the responsible veterinarian, and euthanasia was approved when scores indicated an NHP was terminally ill. Reduced feed consumption, reduced fluid intake, dehydration, no stool production, rough hair coat, 10 to 19% reduction in body weight, nasal discharge, and bleeding at the blood collection site were all assigned 1 point under the clinical scoring system.

Reduced stool production or diarrhea, no fluid intake, weight loss greater than 20%, and bleeding from somewhere other than blood collection site were assigned 2 points.

Temperature changes were scored on a scale of 1 to 3, depending on the severity of the temperature change. Responsiveness was scored on a scale of slightly diminished general activity or subdued (1 point), withdrawn and reduced response to external stimuli (2 points), moderate to dramatically reduced response to external stimuli (e.g., prostrate but able to rise if stimulated) (8 points), and severely or completely unresponsive (e.g., persistently prostrate) (15 points [immediate euthanasia]). Petechia was scored on a scale of: mild (1 point), moderate (2 points), or severe (3 points). Labored breathing was assigned 3 points and agonal breathing 8 points. Additional euthanasia criteria included a combination of severe petechia or bleeding from any orifice, complete anorexia for 24 h, temperature change of greater than 5°F from baseline, moderate to severe depression (determined by evaluating responsiveness), respiratory distress, thrombocytopenia, or severe elevation of gamma glutamyl transferase (GGT), alanine aminotransferase (ALT), alkaline phosphatase (ALP), or blood urea nitrogen (BUN).

### Cells and virus.

Vero E6 cells (Vero C1008, African green monkey kidney origin; BEI Resources catalog no. NR-596) were grown in minimum essential medium (MEM; Gibco) containing 2 mM l-glutamine (Gibco) and 1 mM sodium pyruvate (Gibco) (henceforth referred to as normal growth medium) with 10% heat-inactivated fetal calf serum (FCS; Gibco) at 37°C with 5% CO_2_. The starting virus material consisted of Marburg virus *Homo sapiens*-tc/AGO/2005/Angola-0501379 (species *Marburg marburgvirus*), passage 2 (P2) on Vero E6 cells, acquired from T. Ksiazek, University of Texas Medical Branch. To generate the P3 material, virus was amplified one time using the following method. Vero E6 cells were infected at a multiplicity of infection of 0.001 in normal growth medium containing 2% FCS. Viral supernatant was harvested and clarified by low-speed centrifugation when the cells exhibited 3^+^ cytopathic effects (defined as observing greater than one large patch of cytopathic effects per field when viewed at 4× magnification). Virus was aliquoted and stored in the vapor phase of liquid nitrogen in a cryotank for future use. To generate the P13 material, the P3 virus was amplified 10 additional times, as described above. The cell culture passage as described above was repeated for five passages at three different MOI (0.001, 0.01, and 0.1). This set of passages focused on comparing different MOI and was stopped at passage 5 because data from the first experiment suggested that the rate of adaptation at an MOI of 0.001 had approached a plateau after five passages.

### Determination of viral titers.

Viral titers were determined by plaque assay using an agarose and neutral red assay (previously described in reference 43). Briefly, Vero E6 cells were seeded at a density of 9 × 10^5^ cells per well in 6-well tissue culture plates in normal growth medium with 10% heat-inactivated FCS. The next day, serial dilutions of virus were prepared in normal growth medium containing 2% FCS, media were removed from plates, and 400 µl of each dilution was added to the corresponding well. The plates were incubated for 1 h at 37°C with 5% CO_2_ with constant rocking. After incubation, media were removed from the wells, and a 2-ml primary overlay was added. The primary overlay consisted of 1% Seakem agarose (Lonza) mixed 1:1 with 2× Eagle’s MEM (EMEM; Lonza) containing 4 mM l-glutamine, 2 mM sodium pyruvate, and 4% FCS. Cells were incubated at 37°C with 5% CO_2_ for 7 days. On day 7, 2 ml of secondary overlay was added to each well; the secondary overlay consisted of 1% SeaKem agarose (Lonza) mixed 1:1 with 2× EMEM (Lonza) containing 4 mM l-glutamine, 2 mM sodium pyruvate, 4% FCS, and 8% neutral red solution (Gibco). Cells were incubated at 37°C with 5% CO_2_ for 1 day, and then cell monolayers were inspected for plaques to determine a final titer.

### Electron microscopy.

The electron microscopy studies were performed using a JEOL 100CX transmission electron microscope. Transmission electron microscopy (TEM) grid preparation was carried out in the biosafety level 4 (BSL4) laboratory, as previously described (14). Briefly, viral supernatant was combined with an equal volume of polystyrene bead suspension (Duke Standards 3K/4K series particle counter standards), and the mixtures were deposited onto carbon-Formvar-coated 300-mesh grids and allowed to dry before fixation with 2% glutaraldehyde for 20 min. Grids were rinsed and sterilized via exposure to 1% osmium tetroxide fumes for 1 h before being transferred to the BSL2 laboratory. Grids were removed from the osmium tetroxide, rinsed, and stained with 1% uranyl acetate. During TEM examination, the edges of 10 random grid squares were imaged, and all viral particles and beads were counted. The following equation was used to determine the particle count: [(polystyrene beads counted)/(1 × 10^9^ beads per ml)] = [(viral particles counted)/(actual no. of viral particles present per ml)]. The calculated number of viral particles per milliliter was divided by the number of PFU per milliliter to yield particle/PFU ratios.

### Deep sequencing and sample preparation.

An aliquot of viral supernatant was diluted in TRIzol LS reagent (Ambion) and transferred to the BSL2 laboratory, where RNA was extracted following the manufacturer’s instructions. Following RNA harvest, DNA, rRNA, and mRNA were removed as previously described (44). RNA libraries were then prepared using Illumina’s TruSeq total RNA sample preparation kit, according to the manufacturer’s instructions, as previously described (12, 44). Data analysis began by using the Illumina pipeline to generate a FASTQ file containing all the reads, which was then mapped to the parental virus sequence using Lasergene SeqMan NGen (DNASTAR). The abundance of each nucleotide at each position in the genome was determined and compared to a reference sequence. Changes were then presented as single nucleotide polymorphism (SNP) percentage. Only SNPs occurring at higher than 10% abundance were included in the analysis.

### Experimental inoculation of cynomolgus macaques with MARV.

Sixteen cynomolgus macaques (*Macaca fascicularis*) were intramuscularly (i.m.) injected in the deltoid muscle with 0.5 ml of a target dose of either 0.01 PFU (*n =* 4) or 100 PFU (*n =* 4) of MARV that was either low passage number (P3; *n =* 4) or high passage number (P13; *n =* 4). Eight male and eight female NHPs, 3 to 6 years of age and 2.5 to 5 kg in weight, were used. Nonhuman primates were acquired from Covance (Covance obtained NHPs from an exporter in Vietnam) and serum tested to ensure no reactivity to filovirus antigen prior to purchase (VRL Laboratories, San Antonio, TX). Nonhuman primates were shipped directly to the Texas Biomedical Research Institute for a standard quarantine period. This quarantine period allowed for NHP health status to be evaluated and time for NHPs to acclimate to caging and diet. MARV exposure occurred on study day zero. Blood samples were collected on days 0, 3, 5, 7, and 14 postexposure for analysis of serology, hematology, clinical chemistry, coagulation parameters, and viral load determination. During each scheduled blood collection, rectal temperature was taken and weight was recorded. When animals met euthanasia criteria or at the scheduled end of the project (21 days postexposure), NHPs were euthanized, necropsy was performed, and gross pathological findings were noted. Samples of liver, spleen, heart, kidney, skin, intestine, lymph node, adrenal gland, lungs, and any gross lesions were aseptically removed and divided (with exception of lymph nodes, which remained intact) for viral load determination or fixed in 10% neutral phosphate-buffered (pH 7.2) formalin, processed routinely, embedded in paraffin, sectioned at 5 µm, stained with hematoxylin and eosin, and analyzed.

### Hematology, coagulation, and blood chemistry.

Biochemical analysis was performed using the mammalian liver enzyme profile rotor on a Vet Scan analyzer (Abaxis, Inc.). Complete blood counts were performed using a Vet HM2 machine (Abaxis, Inc.). Coagulation times were determined using the IDEXX Coag Dx analyzer (IDEXX Laboratories).

### Statistics.

The log-rank Mantel-Cox test was used to analyze the survival curves. Two-way ANOVA Bonferroni posttest was used to analyze serum titer, blood chemistry, hematology, and coagulation data.
